# Effect of Channel Height on CO_2_-to-CH_4_ Reduction in Microchannel Electrocatalysis

**DOI:** 10.3390/mi17020148

**Published:** 2026-01-23

**Authors:** Zheng-Yan Lei, Nguyen Van Toan, Masaya Toda, Ioana Voiculescu, Takahito Ono

**Affiliations:** 1 Department of Mechanical Systems Engineering, Tohoku University, Sendai 980-8579, Miyagi, Japan; lei.zheng-yan.q6@dc.tohoku.ac.jp (Z.-Y.L.);; 2 Mechanical Engineering Department, The City College of New York, New York, NY 10031, USA; 3 Micro System Integration Center (μSiC), Tohoku University, Sendai 980-8579, Miyagi, Japan

**Keywords:** CO_2_ reduction, electrocatalysis, microchannel, CO_2_ mass transfer, channel height, metal-assisted chemical etching

## Abstract

Electrocatalytic CO_2_ reduction is a promising approach to mitigate rising atmospheric CO_2_ levels while converting CO_2_ into valuable products such as CH_4_. Conversion into other useful substances further expands its potential applications. However, the efficiency of the CO_2_ reduction reaction (CO_2_RR) is strongly influenced by device geometry and CO_2_ mass transfer in the electrolyte. In this work, we present and evaluate microchannel electrocatalytic devices consisting of a porous Cu cathode and a Pt anode, fabricated via metal-assisted chemical etching (MACE). The porous surfaces generated through MACE enhanced reaction activity. To study the impact of the distance between electrodes, several devices with different channel heights were fabricated and tested. The device with the highest CH_4_ selectivity had a narrow inter-electrode gap of 50 μm and achieved a Faradaic efficiency of 56 ± 11% at an applied potential of −5 V versus an Ag/AgCl reference electrode. This efficiency was considerably higher than that of the device with larger inter-electrode gaps (300 and 480 μm). This reduced efficiency in the larger channel was attributed to limited CO_2_ availability at the cathode surface. Bubble visualization experiments further demonstrated that the electrolyte flow rate had a strong impact on supplied CO_2_ bubble morphology and mass transfer. At a flow rate of 0.75 mL/min, smaller CO_2_ bubbles were formed, increasing the gas–liquid interfacial area and thereby enhancing CO_2_ dissolution into the electrolyte. These results underline the critical role of electrode gap design and bubble dynamics in optimizing microchannel electrocatalytic devices for efficient CO_2_RR.

## 1. Introduction

CO_2_ reduction is a promising strategy to mitigate rising atmospheric CO_2_ concentrations while simultaneously producing value-added products such as CH_4_, CO, C_2_H_5_OH, and HCOOH. Several approaches have been explored for CO_2_ conversion, including thermocatalytic, photocatalytic, and electrocatalytic methods [1,2]. The thermocatalytic method has been shown to exhibit high conversion efficiency and product selectivity; however, it requires a greater energy input to achieve the reaction at elevated operation temperatures and pressures. The photocatalytic method is an environmentally friendly process that applies solar energy to achieve CO_2_ reduction. However, issues such as a lack of product selectivity and conversion efficiency are significant challenges [3,4]. Among these, electrocatalysis is particularly attractive because it operates under ambient conditions and uses water in the electrolyte as the proton source, thereby eliminating the need for H_2_ gas feed and providing a safer and more practical route [5,6,7]. In the electrocatalytic CO_2_ reduction reaction (CO_2_RR), CO_2_ is converted into a range of intermediates that can follow distinct pathways to yield different final products [8,9]. Product distribution in CO_2_RR is highly sensitive to the nature of the electrocatalyst as well as to the local electronic and geometric environment at the electrode surface [10,11,12]. Selective conversion of CO_2_ into CH_4_ is of particular importance because methane is both a high-energy-density fuel and an essential feedstock for the chemical industry. Achieving high selectivity requires careful selection of electrocatalytic materials because their electronic and geometric structures influence product selectivity [9,13,14,15]. To improve product selectivity, various strategies have been used, including atomic-level alloying [16], doping [17], introduction of morphological defects [18,19], and the development of composite materials [20], among others.

We previously proposed a microchannel-based CO_2_ reduction device using porous Cu electrocatalysts to achieve high selectivity and demonstrated its proof of concept [21]. However, further improvement of conversion efficiency requires a deeper understanding of the role of microchannel architecture. In this work, we present a novel microchannel electrocatalytic device designed to investigate the influence of device geometry on the efficiency of CO_2_-to-CH_4_ conversion. The device incorporates a porous Cu cathode and a Pt anode, both fabricated using metal-assisted chemical etching (MACE). Cu-based materials preferentially reduce CO_2_ into hydrocarbons [9,22], while the Pt electrode provides stability by preventing its own oxidation during CO_2_RR. The microfabricated platform further offers scalability for increasing gas processing volumes and facilitates defect engineering of the electrodes [23]. Serpentine device design employs high electrolyte and CO_2_ flow rates over the cathode surface. This high velocity ensures that the gas product, CH_4_, is rapidly swept off the electrode and out of the reaction zone, dramatically reducing its residence time. Consequently, the CH_4_ concentration reaching the Pt anode is exceptionally low, effectively suppressing the unwanted oxidation of CH_4_ and maintaining product integrity. To improve CO_2_ conversion efficiency, the electrode morphology was engineered to be porous through the application of MACE [24,25,26]. In addition, the membrane-less cell design offers significant advantages by reducing internal resistance and ohmic losses, thus improving energy efficiency. It also simplified the structure and reduced manufacturing costs, attributable to the elimination of costly membrane materials, which are further advantages. It is essential to note that this design can circumvent performance degradation induced by membrane fouling or breakdown from reaction by-products.

In addition to the choice of materials, external factors play a crucial role in CO_2_RR. Parameters such as applied potential, ambient temperature, and CO_2_ flow rate in flow-cells considerably influence the conversion efficiency [27,28,29,30], with different operating conditions leading to distinct outcomes. Channel design is also critical because it governs residence time and diffusion distance through adjustments in channel dimensions [31]. For instance, serpentine channels can enhance convection between CO_2_ and the electrolyte [21]. The utilization of microchannels is highly suitable for the study of catalytic characteristics, performance, and reaction mechanisms [32,33]. The high surface-to-volume ratio of microchannels has been demonstrated to be conducive to reaction. The efficiency of the electrochemical reaction can be enhanced through the implementation of precise flow control mechanisms [34]. The utilization of microchannels has also been demonstrated to enhance mass transfer efficiency, thereby augmenting the reaction rate. It is evident that an increase in the presence of bubbles and liquid slugs within the channel concomitantly results in an enhancement of the efficiency of product formation [35,36]. In microdevices, efficient use of space through optimized channel architecture can further maximize conversion efficiency, which can be achieved by combining serpentine layouts with tailored channel heights.

During the experimental process, CO_2_ mass transfer to the cathode surface is a critical factor, governed by CO_2_ solubility in the electrolyte, CO_2_ supply, and the properties of the electrocatalyst [37,38]. It is widely accepted that dissolved CO_2_ serves as the actual reactant in electrocatalytic CO_2_RR [37,39]. Therefore, increasing CO_2_ solubility in the electrolyte can considerably improve conversion efficiency [40]. Solubility is strongly influenced by electrolyte composition, with aqueous and non-aqueous systems exhibiting distinct behaviors [41]. In conventional systems, CO_2_ depletion in the electrolyte often promotes the competing hydrogen evolution reaction (HER), thereby reducing both selectivity and conversion efficiency for CH_4_ production. The microchannel architecture provides a distinct advantage by facilitating rapid CO_2_ diffusion and dissolution, thereby ensuring a sustained CO_2_ supply at the electrode surface and supporting higher CH_4_ conversion efficiency. Therefore, increasing CO_2_ mass transfer is considered a key strategy to improve CO_2_RR performance. Channel design, including parameters such as electrode gaps, directly influences CO_2_ transport dynamics. Among the available measurement approaches, bubble and liquid slug length analysis is commonly used to quantify CO_2_ mass transfer in microchannels. By monitoring bubble length variations along the channel and measuring the associated pressure drop, the rate of CO_2_ mass transfer can be determined [42,43].

In this work, we developed an electrocatalytic device with a novel architecture for converting CO_2_ into CH_4_ and investigated the effect of the distance between electrodes on performance. The device features porous Cu and Pt electrodes, fabricated via MACE. CO_2_ reduction to CH_4_ was performed in a serpentine channel patterned between the top and bottom electrodes using photolithography. The serpentine configuration increases the three-phase contact area between CO_2_ gas, the electrolyte, and the electrodes, thereby enhancing electrocatalytic activity. Additionally, the effects of electrolyte flow rate and CO_2_ bubble size on conversion efficiency were systematically studied.

## 2. Materials and Methods

### 2.1. Device Design

The microdevice was constructed using a three-electrode configuration. The cathode was fabricated via Cu electroless plating, while the anode was prepared by Pt sputtering. Cu was selected as the catalyst owing to its suitability for CH_4_ production in CO_2_RR [22,37], and Pt was used as the anode due to its high anodic stability during the reaction. An Ag/AgCl electrode served as the reference to stabilize the potential. The serpentine channel had a total length of 115 mm and a width of 0.5 mm, with the channel height adjusted to different dimensions by inserting a spacer between the two electrodes. The bonding material used in this work was a dry film photoresist (TMMF NA1000HQ3, Tokyo Ohka Kogyo Co., Ltd., Kawasaki-shi, Japan), selected for its strong adhesion and ability to bond at relatively low temperatures. Silicon wafers of varying thicknesses were used as spacers to adjust the channel height and effectively control the distance between the electrodes. The relationship between channel height and CO_2_ conversion efficiency was systematically evaluated. Additionally, the interaction between CO_2_ and the electrolyte was recognized as a key factor influencing conversion efficiency; higher CO_2_ mass transfer was found to enhance performance [37,39]. Therefore, this study focused on optimizing both channel height and CO_2_ mass transfer to improve CO_2_RR efficiency.

### 2.2. Fabrication Process

**Figure 1:**
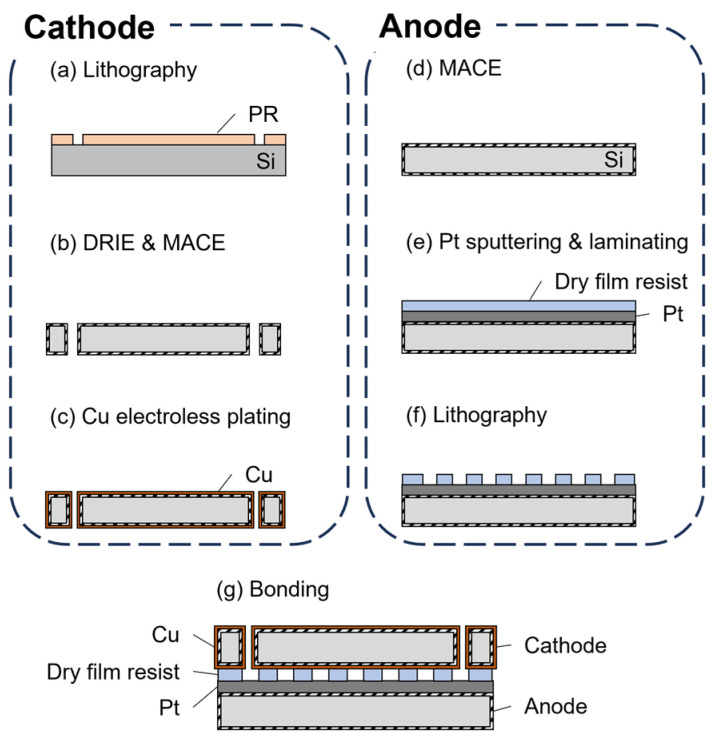
Fabrication process of the microdevice for the CO_2_RR. The cathode was prepared from Si covered with Cu. (**a**) Lithography process to make a pattern on the substrate. (**b**) Inlet and outlet openings were etched in the Si wafer using DRIE and MACE. (**c**) Cu layer was deposited on the substrate by electroless plating. The anode was prepared from Si covered with Pt, and (**d**) the MACE process was applied to produce porous structures. (**e**) Then, after Pt sputtering, (**f**) a dry film photoresist was laminated, and serpentine channel was formed by photolithography. (**g**) The device was assembled by placing the cathode on top of the anode and bonding them by pressing at 150 °C.

The fabricated microdevice comprised a Cu cathode and a Pt anode, bonded using the dry film photoresist. The fabrication process is shown in Figure 1. A Si wafer with a thickness of 380 μm was cut into squares with the dimensions 20 mm × 20 mm. For the cathode fabrication, a positive photoresist (OFPR-800LB 200 cp, Tokyo Ohka Kogyo Co., Ltd., Kawasaki-shi, Japan) was coated on the Si substrate. Circular openings were then patterned using photolithography followed by deep reactive-ion etching (DRIE). To create a porous surface, the substrate underwent a MACE process at room temperature, beginning with the deposition of silver (Ag) nanoparticle catalysts onto the Si substrate for 5 min. During the MACE process, the HF and H_2_O_2_ solution etches the Si substrate underneath the metal catalyst via a localized electrochemical reaction. The etchant was mixed with H_2_O_2_, HF, and DI water at a ratio of 2:5:1. Metal nanoparticles both etch and migrate into Si, forming small voids and producing a porous surface. This porous structure is crucial for subsequent Cu electroless plating on the cathode. First, the porous Si substrate was sensitized with SnCl_2_ and activated with PdCl_2_ (Tin(II) Chloride and Palladium(II) Chloride, FUJIFILM Wako Pure Chemical Corp., Osaka, Japan) at room temperature for 4 min. Then, the substrate was placed in a Cu solution (OPC Copper NCA-1, Okuno Chemical Industries Co., Ltd., Osaka, Japan) at 60 °C for 20 min. For the anode, a porous Si structure was also fabricated using MACE, followed by sputtering a 32 nm-thick Ti layer as an adhesion layer and a 175 nm-thick Pt layer to serve as the anode. Sputtering was performed at room temperature under a pressure of 0.5 Pa and a power of 300 W. Subsequently, a 50-μm-thick dry film photoresist was laminated onto the surface. The serpentine channel was then patterned using UV photolithography. Finally, the two electrodes were bonded together using a bonder (EVG520, EV Group, St. Florian am Inn, Austria) at 150 °C under a pressure of 10 N for 15 min.

**Figure 2:**
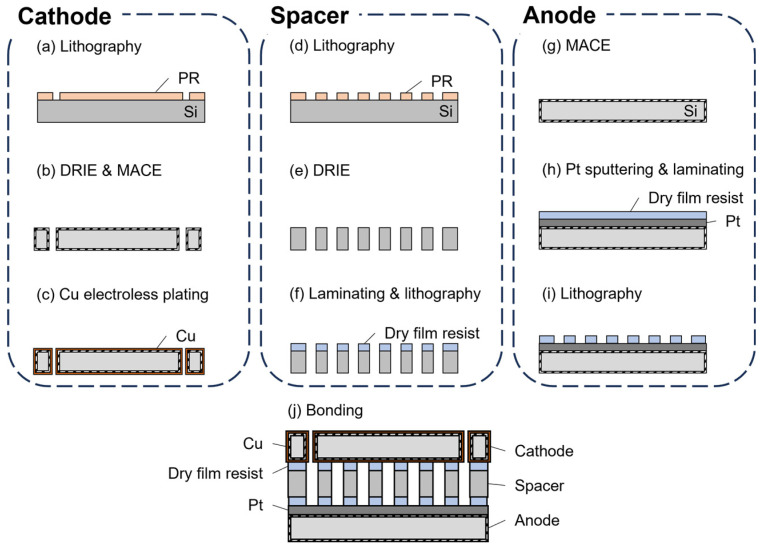
Fabrication process of a microdevice with a Si spacer for the CO_2_RR. The processes of the cathode (**a**–**c**) and the anode (**g**–**i**) were the same as in Figure 1. (**d**) The spacer was fabricated from a Si substrate and (**e**) etched by DRIE to form a serpentine channel. (**f**) Then, a layer of dry film resist was laminated as a bonding material. (**i**) Photolithography was done for forming the serpentine channel. (**j**) The device was assembled by using the spacer as an adhesive in the middle of the cathode and anode at 150 °C.

To adjust the channel height, Si substrates with thicknesses of 200 μm or 380 μm were used as spacer layers between the electrodes. As shown in Figure 2, serpentine channels were patterned on the spacer substrates using photolithography and DRIE. A dry film photoresist was then laminated onto the surface to serve as an adhesion layer for bonding with the electrodes. The device was assembled by placing the anode at the bottom, the spacer in the middle, and the cathode on top, followed by bonding at 150 °C under a force of 10 N for 15 min. Devices with channel heights of 50, 300, and 480 μm were fabricated. After bonding, electrical connections were established by attaching Cu wires to each electrode using conductive epoxy (EPO-TEK H20E, Epoxy Technology, Inc., Billerica, MA, USA). Four commercial tube connectors were glued in alignment with the circular openings, and an Ag/AgCl reference electrode was installed in one of the connectors to stabilize the potential.

### 2.3. Measurement Setup

**Figure 3:**
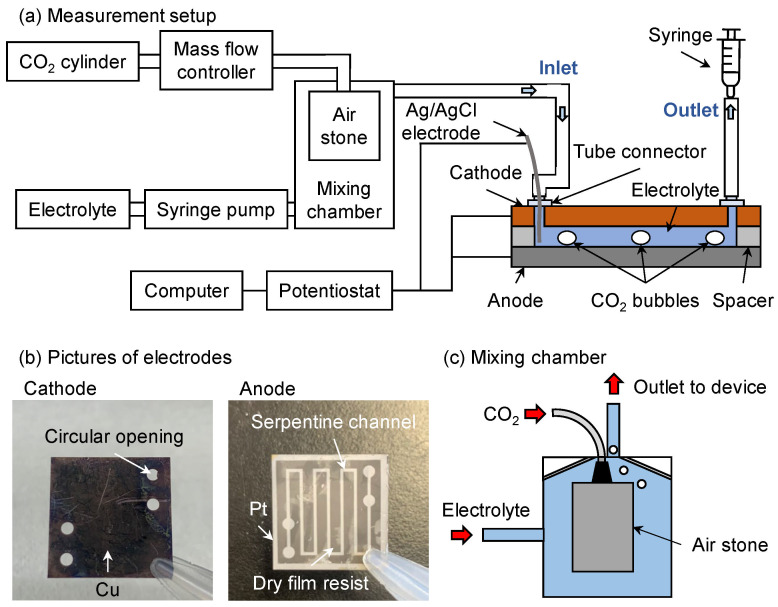
(**a**) Schematic of the measurement setup. CO_2_ and the electrolyte were mixed and introduced through the inlet. An air stone was used to generate bubbles, enhancing the gas–liquid interface. The anode and cathode of the microdevice were connected to a potentiostat together with an Ag/AgCl reference electrode. (**b**) Photographs of the two electrodes. Circular openings on the cathode served as the inlet and outlet of the microdevice, while the serpentine channel patterned in the dry film photoresist is visible on the anode. (**c**) Illustration of the mixing chamber showing the air stone device and the directions of fluid flow.

For measurements, CO_2_ gas and the electrolyte were first introduced into a mixing chamber to achieve initial homogenization. The resulting mixture then flowed through the microdevice and was collected using a syringe, as shown in Figure 3. High-purity CO_2_ gas (99.99%) was supplied at a flow rate of 1 sccm from a gas cylinder and controlled via a mass flow controller. The electrolyte consisted of 0.5 M NaHCO_3_ with 0.1 vol% HCOOH in deionized water and was delivered at a flow rate of 0.5 mL/min using a syringe pump. The purpose of adding HCOOH to the electrolyte is to suppress the Cu oxidation. The mixing chamber measured 22 × 22 × 30 mm^3^ and contained an air stone to facilitate gas–liquid mixing. The air stone generated small CO_2_ bubbles, increasing the interfacial area between the gas and electrolyte. A three-electrode potentiostat (HA151B, Hokuto Denko Co., Tokyo, Japan) was used to apply the anode–cathode potential to the microdevice. The collected gas samples were analyzed by gas chromatography (GC-14B, Shimadzu, Kyoto, Japan) equipped with a SHINCARBON-ST Φ3 mm stainless steel column. The column temperature was ramped from 40 °C to 200 °C at 20 °C/min, with helium serving as the carrier gas. A data logger recorded peak heights and areas during the experiment, from which the Faradaic efficiency for CH_4_ production was calculated [44].

**Figure 4:**
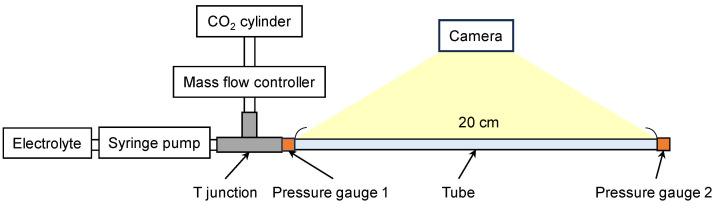
Schematic of the measurement setup for observing CO_2_ bubble formation and size changes in the electrolyte in the tube. The CO_2_ cylinder was connected to a mass flow controller to maintain a constant gas flow rate, while the electrolyte was injected using a syringe pump. Pressure gauges were installed at the inlet and outlet to monitor the pressures in the tube. A camera recorded bubble size variations to evaluate CO_2_ gas diffusion into the electrolyte.

To measure CO_2_ mass transfer at different electrolyte flow rates, the experimental setup shown in Figure 4 was constructed. CO_2_ flow was controlled at 1 sccm using a mass flow controller, while the electrolyte flow rate was varied between 0.1 and 1 mL/min using a syringe pump. A T-junction connected the syringe pump and mass flow controller, allowing CO_2_ and the electrolyte to flow into the circular tube. The tube had a total length of 20 cm and an inner diameter of 2 mm. A camera was used to record and observe CO_2_ bubble behavior and deformation in the tube. The pressures at the inlet and outlet were measured using a pressure gauge (GC04-174, Nagano Keiki Co., Ltd., Tokyo, Japan). The outlet was open-ended; thus, its pressure was approximately atmospheric. CO_2_ bubble sizes were analyzed using the open-source software ImageJ 1.54g (NIH, Bethesda, MD, USA), with a vernier caliper serving as a reference for actual dimensions. The CO_2_ mass transfer rate was then determined based on the measured bubble sizes and the pressure difference.

## 3. Results

**Figure 5:**
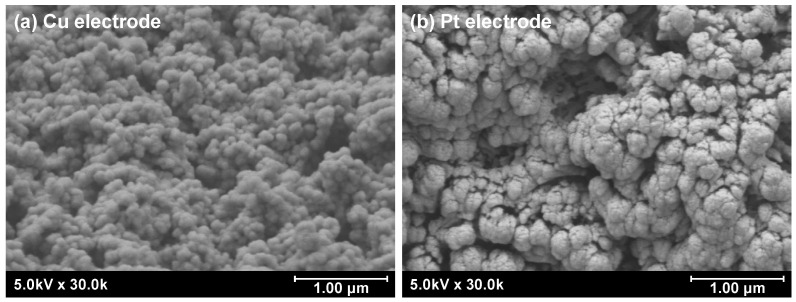
SEM images of the electrodes after (**a**) Cu electroless plating and (**b**) Pt sputtering.

**Figure 6:**
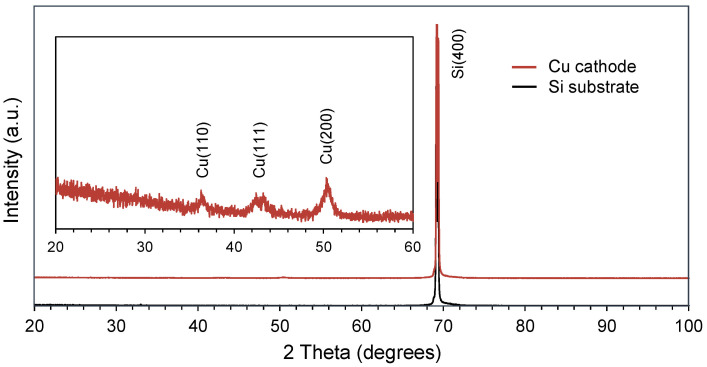
The result of the XRD analysis of the Cu catalyst surface.

Scanning electron microscopy (SEM; SU-70, Hitachi High-Tech, Tokyo, Japan) was used to observe the surface of the electrodes. The original surface of the Si substrate was smooth. After undergoing the MACE process, however, the surface of the electrodes became porous, as shown in Figure 5. This nanostructure increased the surface area when metal deposition was applied. The porous surface structure of the substrate was maintained after the Cu electroless plating and Pt sputtering. This increased the number of active sites and promoted the reaction. In addition, an XRD analysis (D8 ADVANCE, Bruker Corp., Billerica, MA, USA) was applied to measure the surface crystal facet of the Cu catalyst, as shown in Figure 6. Because the surface of the Cu catalyst was a porous nanostructure, the detected crystal facets also exhibit a polycrystalline appearance.

**Figure 7:**
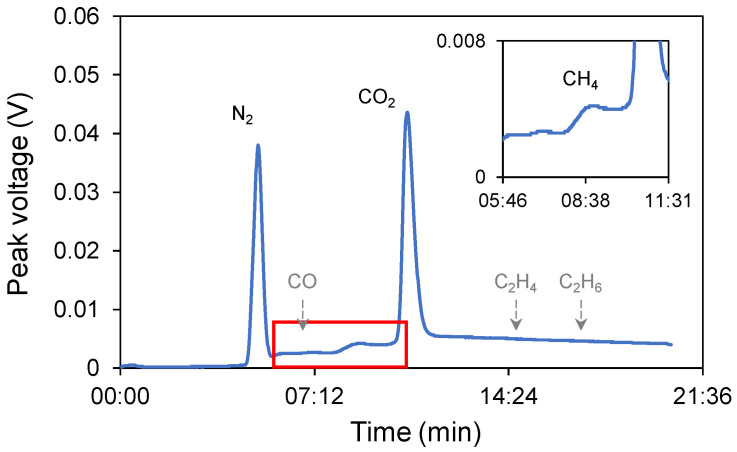
The result of analyzing the GC of the gas sample upon the application of −5 V. The inset is an enlarged view of the area within the red frame.

The measurement result is shown in Figure 7. The retention times of gas components in the GC chromatogram are pointed out in the figure. It was evident that only N_2_, CH_4_, and CO_2_ had an obvious peak during the experiments. The column can still analyze CO, C_2_H_4_, and C_2_H_6_ (colored in grey), yet these compounds were not detected in the product. This finding serves to substantiate the device’s product selectivity. In the present system, CH_4_ generated at the cathode could, in principle, undergo oxidation at the Pt anode. However, several factors indicate that this effect is negligible under our experimental conditions. First, CH_4_ has an extremely low solubility in water (Henry’s constant ≈ 1.4 × 10^−3^ mol L^−1^ atm^−1^ at 25 °C), corresponding to a dissolved concentration below 10^−5^ mol L^−1^ in the electrolyte. In addition, the high electrolyte flow rate (0.5 mL/min) and short inter-electrode distance (50 µm) rapidly sweep CH_4_ bubbles away from the cathode, minimizing gas crossover to the anode. Gas-chromatographic analysis detected only CH_4_ and CO_2_ peaks within the 1 ppm detection limit, and no CO or other oxidation products were observed. These results suggest that any CH_4_ oxidation at the Pt anode, if present, is below the experimental uncertainty and does not influence the calculated Faradaic efficiency.

**Figure 8:**
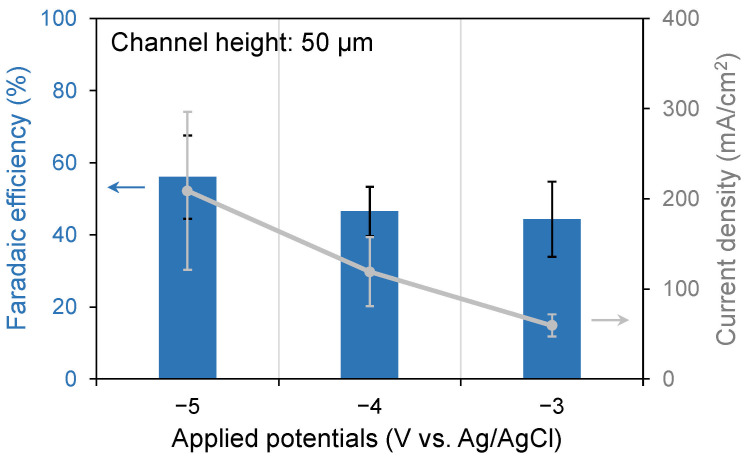
Dependence of FE and current density on the applied potential. The channel height of the device was 50 μm.

The present study examined the dependence of FE and current density on potential by applying a potential ranging from −3 to −5 V vs. Ag/AgCl, as shown in Figure 8. The device with a 50 μm channel height was utilized in this experiment. The CO_2_ flow rate was 1 sccm, and the electrolyte was 0.5 M NaHCO_3_ mixed with 0.1 vol% HCOOH. The current density exhibited an increase in proportion to the potential. The highest FE and current density were observed at an applied potential of −5 V. At this potential, the current density was high, which resulted in a high dissociation degree of the solution and the promotion of the reduction reaction.

**Figure 9:**
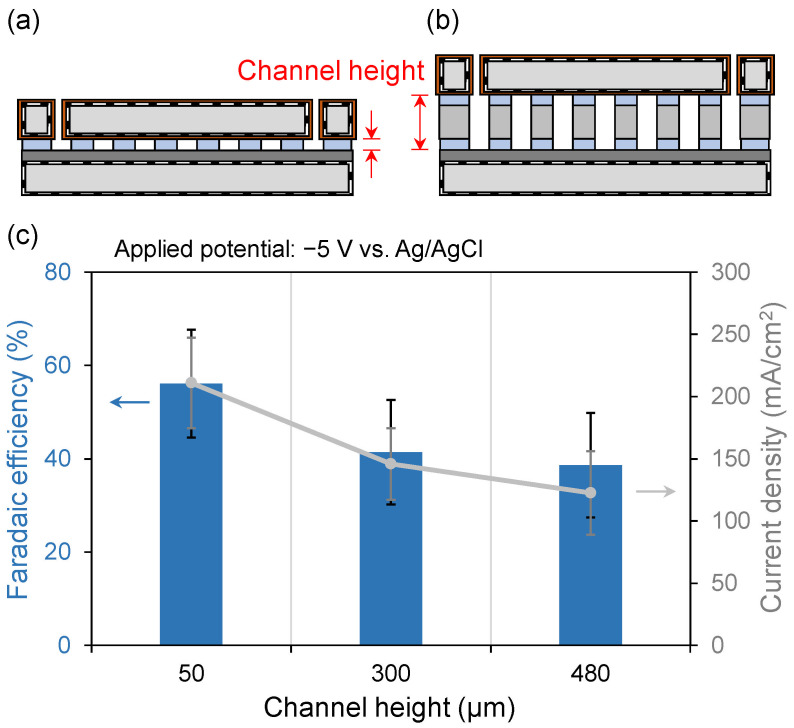
Relationship between channel height and Faradaic efficiency at an applied potential of −5 V on the microdevice. (**a**) Device with 50 μm height of the serpentine channel fabricated in the dry film photoresist. (**b**) Devices with serpentine channels of 300 and 480 μm heights fabricated using Si spacers as intermediate layers. (**c**) The 50 μm channel height device exhibited the highest average Faradaic efficiency of 56.1%. As shown in (**c**), Faradaic efficiency decreased as the channel height increased.

The channel height adjustment experiment was performed using a dry film resist and Si wafers of varying thicknesses. The relationship between channel height and CO_2_ conversion efficiency is shown in Figure 9. A device with a 50 μm channel height is shown in Figure 9a, while devices with 300 and 480 μm channel heights are depicted in Figure 9b. The applied potential was −5 V vs. Ag/AgCl, and the CO_2_ flow rate was maintained at 1 sccm. As shown in Figure 9c, the device with the smallest channel height of 50 μm exhibited the highest Faradaic efficiency of 56.1%. In comparison to the devices with 50 μm and other high channel devices, the presence of larger bubbles in the higher channel may affect the local CO_2_ mass transfer process. CO_2_ is replenished via HCO_3_^−^ equilibrium [45]; the stagnant flow conditions associated with larger bubbles could potentially reduce the effective turbulence near the cathode surface compared to smaller, faster-moving bubbles in a confined narrow channel. As a result, CO_2_ that did not react at the cathode surface was carried directly to the outlet, reducing the overall conversion efficiency compared to devices with smaller channel heights. The microdevice with a 50 μm channel design provides significant advantages by simultaneously mitigating both Ohmic and mass transport losses, which is critical for high-performance operation. At higher current densities, the presence of sufficient CO_2_ on the cathode surface has been shown to increase CH_4_ conversion and reduce the probability of HER. In devices with channel heights of 300 and 480 μm, it was observed that the higher the channel height, the larger the CO_2_ bubble volume. Consequently, the capacity for CO_2_ molecules to adsorb onto the cathode surface for reduction is decreased, leading to lower efficiency.

**Figure 10:**
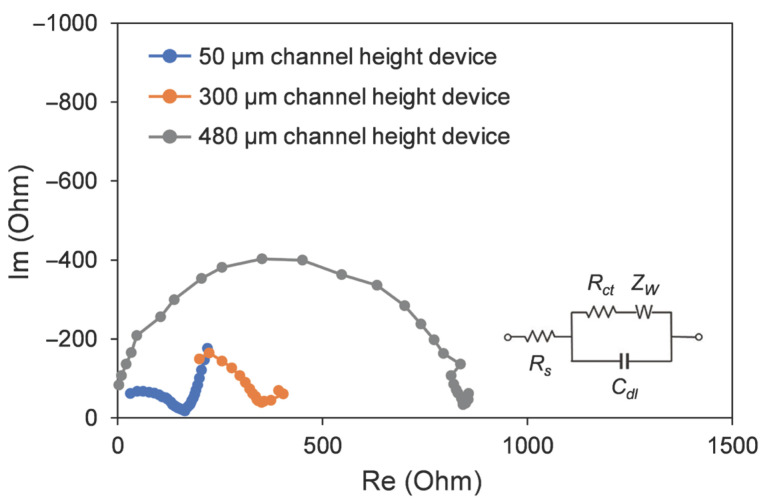
The EIS experiment results of devices with different channel heights.

Devices with varying channel heights were examined in the Electrochemical Impedance Spectroscopy (EIS) experiment, as shown in Figure 10. The blue line indicated a channel height of 50 μm, the orange line indicated a height of 300 μm, and the gray line indicated a height of 480 μm. This analysis revealed a clear correlation between the channel height and the microdevice impedance. The charge transfer resistance (*R_ct_*) was found to be proportional to the channel height. The device featuring the highest channel height (480 µm) exhibited a significantly larger *R_ct_* in comparison to other devices, indicating an overall higher impedance. Conversely, the device with the smallest channel height (50 µm) displayed the lowest impedance. This reduction in impedance suggests a more facile electrochemical reaction kinetics. The reduced channel height minimizes the ohmic resistance (*R_s_*) by decreasing the average path length for ion migration. The employment of a smaller channel height has been demonstrated to engender a decrease in total internal impedance, which, in turn, has been shown to engender a greater current density. Consequently, a higher CO_2_RR conversion efficiency was observed under the same applied potential.

**Figure 11:**
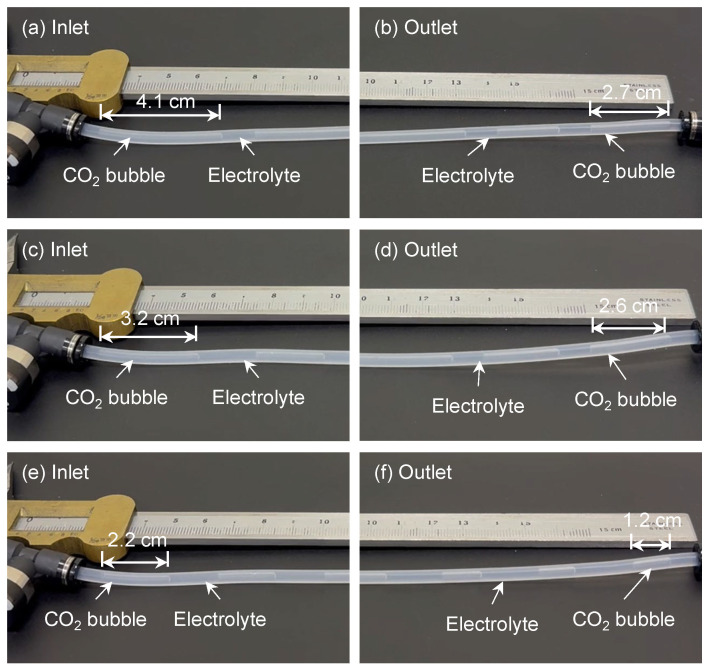
Photographs of the CO_2_ mass transfer experiment under different electrolyte flow rates in the supply tube. The CO_2_ flow rate was maintained at 1 sccm, while the electrolyte flow rates were (**a**,**b**) 0.25 mL/min, (**c**,**d**) 0.5 mL/min, and (**e**,**f**) 0.75 mL/min. The relationship between bubble size and electrolyte flow rate is illustrated, showing that CO_2_ bubbles were larger at lower flow rates and smaller at higher flow rates, such as 0.75 mL/min.

CO_2_ mass transfer, defined as the ability of CO_2_ gas to dissolve into the solution, determines the CO_2_ concentration in the electrolyte and plays a crucial role in electrocatalytic CO_2_RR. Higher mass transfer efficiency can enhance overall reaction efficiency [37,39]. The absorption of CO_2_ in microchannels is predominantly influenced by physical and chemical processes [46]. During physical absorption, CO_2_ molecules that enter the liquid slugs do not disappear. As the mass transfer process continues, CO_2_ in the liquid phase becomes increasingly saturated, leading to a decline in the mass transfer rate. During the process of chemical absorption, CO_2_ is consumed, thereby enabling chemical absorption to maintain a higher rate of mass transfer than physical absorption for a given period of time. The measurement conditions and results are summarized in Table 1. Bubble size analysis was performed using the open-source software ImageJ 1.54g, as shown in Figure 11, with a vernier caliper serving as a reference for accurate measurement. The CO_2_ flow rate was maintained at 1 sccm while varying the electrolyte flow rate to observe changes in bubble size in the tube. At lower electrolyte flow rates, CO_2_ bubbles were longer, whereas higher flow rates generated numerous smaller bubbles. This dependence arises because CO_2_ bubbles experience greater resistance in the faster-moving electrolyte, resulting in smaller bubble formation at higher flow rates. However, at an electrolyte flow rate of 1 mL/min, the increased pressure from the fast-flowing electrolyte hindered gas entry into the tube, making it impossible to observe bubble formation. At an electrolyte flow rate of 0.1 mL/min, the CO_2_ bubbles generated in the tube were larger in volume, making it difficult to observe size changes. Additionally, for all flow rates, bubbles at the inlet were larger than those at the outlet. The bubble volume gradually decreased along the tube owing to mass transfer, because CO_2_ in the gas phase dissolved into the electrolyte and transitioned into the aqueous phase, resulting in a continuous reduction in bubble size.

## 4. Discussion

### 4.1. Effect of Channel Height on Conversion Efficiency

**Figure 12:**
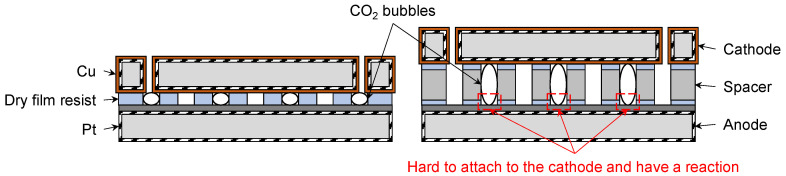
Illustration of cross-sectional views of the microdevice, with and without a spacer layer, showing CO_2_ bubbles flowing through the serpentine microchannel. CO_2_RR occurs at the cathode surface. Devices with larger channel heights allowed the formation of larger CO_2_ bubbles in the channel.

As shown in Figure 9, devices with smaller channel heights exhibited higher CO_2_ conversion efficiencies. The CO_2_RR predominantly occurs at the triple-phase interface of gas, liquid, and cathode [6,47]. In devices with larger channel heights, such as 480 μm, CO_2_ bubbles were larger (Figure 12), which reduced the effective gas–liquid–cathode interfacial area and limited the amount of CO_2_ that could be adsorbed and reduced at the cathode. CO_2_ gas that did not dissolve in the electrolyte passed directly to the outlet, resulting in decreased conversion efficiency owing to the increased fraction of unreacted CO_2_.

The complete electro-reduction of CO_2_ to CH_4_ requires an eight-electron, eight-proton transfer process, as shown in Equation (1):

(1) CO_2_ + 8H^+^ + 8e^−^ → CH_4_ + 2H_2_O.

On copper-based catalysts, this pathway generally proceeds via the initial formation of CO*, which is the key intermediate for all hydrocarbon products. Due to the high negative bias used in this study, the efficient initial activation of CO_2_ to CO* was achieved, thereby facilitating the desired reaction. The porous Cu structure exhibited a strong binding affinity for CO*, thereby suppressing CO desorption and enabling further reduction. The large negative bias provided the driving force to overcome the high activation barrier for the CO* hydrogenation step. This, in turn, accelerated the conversion process towards the final CH_4_ product.

The enhanced Faradaic efficiency observed in devices with narrower inter-electrode gaps can be attributed to the combined effects of chemical equilibria, mass transfer, and the local electric field. At the highly negative applied potential of −5 V, both the CO_2_RR and the competing HER occur at the cathode, resulting in a rapid accumulation of hydroxide ions according to Equation (2).

(2) 2H_2_O + 2e^−^ → H_2_(g) + 2OH^−^.

The increased local OH^−^ concentration shifts the equilibrium of dissolved CO_2_ toward bicarbonate and carbonate species:

(3) CO_2_(aq) + OH^−^ → HCO_3_^−^,

(4) HCO_3_^−^ + OH^−^ → CO_3_^2−^ + H_2_O.

In devices with larger channel heights, such as 480 μm, OH^−^ ions accumulate more readily near the cathode owing to the longer diffusion path, accelerating chemical conversions that deplete the concentration of free CO_2_ available for electroreduction. This CO_2_ starvation at the electrode surface lowers the Faradaic efficiency for methane formation. Conversely, in the 50 μm channel device, OH^−^ ions are transported more efficiently toward the anode, where they participate in oxidation reactions, mitigating excessive alkalization at the cathode. Therefore, the local concentration of free CO_2_ is maintained and favors the electrocatalytic reduction to CH_4_.

Additionally, the shorter inter-electrode distance strengthens the local electric field, facilitating electron transfer and stabilizing ion transport across the channel. Collectively, these factors—suppression of CO_2_ depletion via equilibrium control, enhanced bubble-mediated mass transfer, and stronger electric field effects—synergistically explain why the device with a 50 μm channel height achieved the highest Faradaic efficiency of 56.1% compared with devices having larger inter-electrode gaps.

### 4.2. Gas–Liquid Mass Transfer and Interfacial Behavior

**Figure 13:**
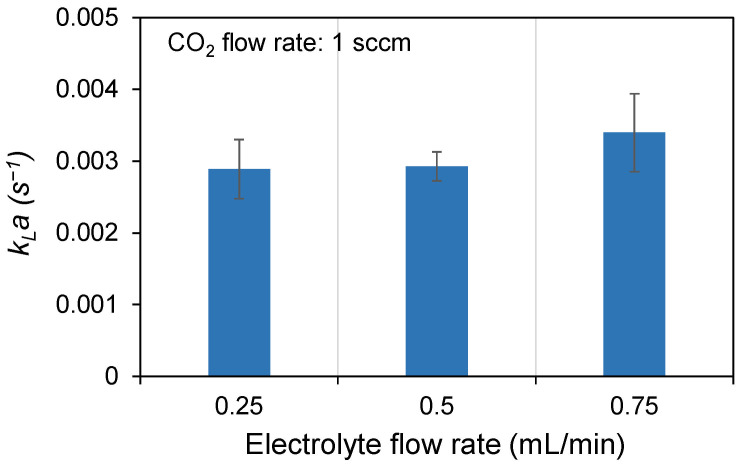
Relationship between electrolyte flow rate and mass transfer (*k_L_a*). Mass transfer was highest at an electrolyte flow rate of 0.75 mL/min due to smaller CO_2_ bubbles, which increased the number of gas–liquid interfaces and enhanced CO_2_ transfer.

As shown in Figure 11, the CO_2_ bubble size is strongly influenced by the electrolyte flow rate. Proper adjustment of the flow rate can generate a larger number of smaller CO_2_ bubbles, increasing the gas–liquid interfacial area and enhancing the dissolution of CO_2_ into the electrolyte slugs. To quantitatively assess CO_2_ mass transfer in the tube, the mass transfer coefficient (*k_L_a*) can be determined using Equations (5) and (6) [41,42,48]:

(5) $$k_{L}a=\frac{\varepsilon }{t}\ln(\frac{C_{e}-C_{0}}{C_{e}-C_{out}}),$$

where *ε* is the fraction of the liquid-phase volume in the tube (%), *t* is the residence time of the fluid in the microchannel (s), and *C_e_*, *C*_0_, and *C_out_* are the CO_2_ concentrations in the liquid phase (mol L^−1^) at equilibrium, at the channel inlet, and at the channel outlet, respectively. *C*_0_ is taken as zero because the initial concentration is negligible. *C_out_* can be calculated as:

(6) $$C_{out}=\frac{\Delta n}{V_{L}}=\frac{P_{0}V_{0}-P_{out}V_{out}}{RT(wdL_{s})},$$

where *P*_0_ and *P_out_* represent the pressures at the inlet and outlet, respectively, *V* is the volume, *w* is the channel width, *d* is the channel depth, and *L_s_* is the length of the liquid slug. The results are shown in Figure 13. CO_2_ bubble deformation was observed at electrolyte flow rates ranging from 0.25 to 0.75 mL/min, with a constant CO_2_ flow rate of 1 sccm. At an electrolyte flow rate of 0.75 mL/min, numerous small bubbles were formed. A greater number of interfaces between bubbles and liquid slugs leads to higher mass transfer. At an electrolyte flow rate of 0.25 mL/min, the CO_2_ bubbles formed were large, reducing the number of gas–liquid interfaces in the tube and consequently decreasing mass transfer. Increasing the electrolyte flow rate enhanced turbulence at the gas–liquid interface, accelerating the surface renewal rate [41]. This improvement in CO_2_ mass transfer subsequently enhanced CO_2_RR activity [40]. Therefore, optimizing the flow rate to generate a larger gas–liquid interfacial area can increase CO_2_ mass transfer and improve overall conversion efficiency.

Furthermore, the narrower channel geometry confines CO_2_ bubbles, producing smaller and more dispersed bubbles that increase the gas–liquid interfacial area and enhance mass transfer, as previously discussed. Smaller bubbles dissolve faster into the electrolyte, improving the supply of CO_2_ molecules to the active sites of the porous Cu cathode. This behavior aligns with the observed higher *k_L_a* at intermediate electrolyte flow rates. The process of bubble formation is influenced by multiple factors, including the dimensions of the channel, the flow of gas and liquid, and the properties of the gas and liquid. Additionally, the mode of contact between the gas and liquid, the flow within the primary or secondary channel, and the configuration of the system play a significant role in determining the outcome. This, in turn, has consequences for mass transfer [49]. Further research is required in order to develop a more comprehensive evaluation and thus a superior design method.

### 4.3. Energy Efficiency and Overpotential

In addition to mass transfer considerations in CO_2_RR, the energy conversion efficiency of the device is also critical. Achieving high conversion efficiency with minimal energy input is essential. The energy conversion efficiency is defined as the ratio of the standard enthalpy of combustion of the produced CH_4_ (890 kJ/mol) to the electrical energy input, calculated as *VIt*, where *V* is the applied potential, *I* is the current, and *t* is the reaction time. For devices with channel heights of 50, 300, and 480 μm at −5 V vs. Ag/AgCl, the energy conversion efficiencies were 32.0%, 20.6%, and 21.2%, respectively. It should be noted that the applied potential of −5 V vs. Ag/AgCl is much more negative than the thermodynamic potential for CO_2_-to-CH_4_ conversion (−0.97 V vs. Ag/AgCl), representing a large overpotential. Such overpotential results in considerable energy losses and promotes side reactions, including hydrogen evolution. The improved efficiency of the 50 μm channel device is attributed not only to enhanced CO_2_ utilization, but also to reduced apparent overpotential losses caused by mass transfer limitations. Optimizing the device is essential to lower energy consumption and increase energy conversion efficiency, for example, by adjusting catalyst morphology, electrolyte usage, channel design, and the microenvironment to facilitate intermediate formation [19]. Various strategies should be considered to enhance both CO_2_ conversion efficiency and overall energy efficiency.

## 5. Conclusions

An electrocatalytic device with a serpentine microchannel was fabricated for CO_2_ reduction to CH_4_, demonstrating good product selectivity. Among devices with different channel heights, the one with the smallest channel height exhibited superior performance, achieving a Faradaic efficiency of approximately 56.1%. Due to the limited cathode surface area, only a finite amount of CO_2_ could react, and excessive CO_2_ reduced the overall conversion efficiency. Moreover, gas–liquid interfacial dynamics significantly influence CO_2_ availability to the cathode, and thereby the CO_2_RR efficiency. Increasing the interfacial area between CO_2_ bubbles and electrolyte slugs enhanced CO_2_ mass transfer. Therefore, careful control of gas and liquid flow rates is essential, because it affects bubble formation and deformation, thereby impacting CO_2_ mass transfer and overall conversion efficiency. Although a device with 50 µm channel height can produce a FE of CH_4_ around 56.1%, further improvements to efficiency remain a key issue. In addition, reducing energy consumption is a potential problem that needs to be solved. When the enlarged-scale design is implemented in the future, conversion efficiency can be increased while reducing energy consumption. Another key point is the long-term use of the CO_2_RR equipment. The issue of how to circumvent problems such as material oxidation and channel contamination to extend the lifetime of the device is a challenge for CO_2_RR technology.

## Figures and Tables

**Table 1:** Relationship between CO_2_ bubble size and tube pressure at the inlet and outlet with electrolyte flow rates from 0.1 to 1 mL/min.

CO_2_ Flow Rate (sccm)	Electrolyte Flow Rate (mL/min)	Inlet Bubble Size (cm)	P_0_ (kPa)	Outlet Bubble Size (cm)	P_out_ (kPa)
1	0.1	Too big bubble size			
	0.25	4.14	0.598	2.71	0.085
	0.5	3.21	0.783	2.56	0.125
	0.75	2.18	1.073	1.17	0.068
	1	No CO_2_ bubbles			

## Data Availability

The original contributions presented in this study are included in the article. Further inquiries can be directed to the corresponding author.
